# Efficacy and safety of oral sulfate tablet vs. polyethylene glycol and ascorbate for bowel preparation in children

**DOI:** 10.3389/fped.2024.1277083

**Published:** 2024-02-08

**Authors:** Sujin Choi, Ji Sook Kim, Byung-Ho Choe, Ben Kang

**Affiliations:** Department of Pediatrics, School of Medicine, Kyungpook National University, Daegu, Republic of Korea

**Keywords:** bowel preparation, oral sulfate tablet, child, efficacy, safety

## Abstract

**Background and aim:**

Bowel preparation for pediatric colonoscopy presents several challenges. However, no bowel preparation regimen is universally preferred for children. We aimed to investigate the efficacy and safety of oral sulfate tablet (OST) in pediatric bowel preparation.

**Methods:**

This study retrospectively analyzed data from children who received 2l of polyethylene glycol and ascorbate (PEG/Asc) or OST for bowel preparation between 2021 and 2023. A comparative analysis was conducted between the two groups.

**Results:**

A total of 146 patients were included (2l PEG/Asc: 115, 73.0% vs. OST: 31). No significant difference was observed in the total BBPS score (median 8.0 vs. 8.0, *P *= 0.152) and the total OBPS score (median 5.0 vs. 3.0, *P* = 0.152) between the two groups. No significant difference was noted in the ratio of a bubble score of 0 (73.0% vs. 93.5%, *P* = 0.132). The incidence of abdominal pain was significantly lower in the OST group (32.2% vs. 3.2%, *P* = 0.002). The VAS score for overall satisfaction was significantly higher in the OST group (4.0 vs. 7.0, *P* < 0.001). For the next colonoscopy bowel preparation, a higher proportion of patients in the OST group showed a willingness to use the same preparation regimen (33.9% vs. 83.9%, *P* < 0.001).

**Conclusion:**

OST was as efficacious and safe as 2 L of PEG/Asc for pediatric bowel preparation. The satisfaction level was higher with OST than with 2 L of PEG/Asc. OST may be considered a good alternative for children with poor compliance during bowel preparation.

## 1 Introduction

The use of pediatric colonoscopy has gradually increased worldwide owing to the increasing incidence of intestinal diseases such as pediatric inflammatory bowel disease (1–5). Adequate bowel preparation is essential for successful pediatric colonoscopy. However, no universally approved bowel preparation regimens have been established for children, and standardized protocols for bowel preparation are lacking.

For bowel preparation in children, recent clinical guidelines recommend low-volume preparation using polyethylene glycol (PEG) along with ascorbate (PEG/Asc) or picosulfate magnesium citrate (6, 7). Children find it challenging to ingest the relatively large volume of preparatory solutions with unpleasant taste, and approximately one-third of pediatric colonoscopies are associated with poor bowel cleansing (7–10). Patient compliance with the regimen is crucial for pediatric colonoscopy; in some cases, poor patient compliance requires the administration of the solution through a nasogastric (NG) tube (11).

To improve compliance with taking bowel preparation solutions, some tableted purgatives have been developed and used in adults (12–17). Among them, oral sulfate tablet (OST) is an attractive agent that addresses the safety concerns of tableted sodium phosphate (NaP), such as renal toxicity, and removes the unpleasant taste of the oral sulfate solution (15–17). Despite reports on the efficacy and safety of OST in adults, no relevant data are available in children. As OST can be an alternative for bowel preparation in children with poor compliance, this study aimed to investigate the efficacy and safety of OST vs. PEG/Asc for bowel preparation in children undergoing elective colonoscopy.

## 2 Material and methods

### 2.1 Patients and study design

This retrospective study analyzed data from children who underwent elective colonoscopy at a children's hospital between January 2021 and May 2023 in South Korea. Patients who had successfully taken 2 L of PEG/Asc or OST for bowel preparation, and those who underwent colonoscopy in the morning were included in the analysis. Patients who had failed bowel preparation and were unable to undergo colonoscopy or those who underwent colonoscopy in the afternoon were excluded. Both groups received a split-dose regimen, with the first and second doses administered in the evening before and the morning of the colonoscopy, respectively. The 2l PEG/Asc group took Coolprep® powder (Taejoon Pharmaceuticals Korea; ascorbic acid, 4.7 g; polyethylene glycol 3,350, 100 g; potassium chloride 1.015 g; sodium ascorbate 5.9 g; sodium chloride 2.691 g; sodium sulfate anhydrous 7.5 g) and the OST group took Orafang® tablets (Pharmbio Korea Inc.; composition: anhydrous sodium sulphate, 1,125 mg; potassium sulphate, 201.07 mg; anhydrous magnesium sulphate, 102.86 mg; simethicone, 11.43 mg) for bowel cleansing. Electronic medical charts were reviewed, and clinical amd laboratory data were extracted. A comparative analysis was conducted between the two groups.

### 2.2 Definitions and outcomes

The primary efficacy endpoint was the overall adequacy of bowel preparation. The Boston bowel preparation scale (BBPS), Ottawa bowel preparation scale (OBPS), and bubble score were investigated. In the BBPS, a total score of 9 indicates excellent preparation, whereas a score of ≥6 indicates adequate preparation. In the OBPS with summative scores of 0–14, a score of 0 indicates excellent preparation. The bubble score ranges from 0 to 3, with 0 indicating minimal or no bubbles. The secondary efficacy endpoint was the patient's satisfaction and tolerability. The ease-of-use and taste scores were analyzed. The 10-level visual analog scale (VAS) scores were reviewed for overall satisfaction. For the safety assessment, adverse events and laboratory test results for electrolyte levels and renal function were analyzed.

### 2.3 Statistical analysis

To compare the fluctuations over the year, a 95% confidence interval (CI) was calculated using the Clopper-Pearson method (14). For statistical comparison between two groups, chi square test or Fisher's exact test was used for categorical variables, while Student's t test were used for statistical comparison of continuous variables. Comparative data for continuous variables are expressed as medians with interquartile range (IQR) or means with standard deviation (SD). Data were considered to be statistically significantly different if *P* < 0.05. Statistical analyses were conducted using R version 3.2.3 (http://www.r-project.org).

### 2.4 Ethics statement

This study was approved by the Institutional Review Board (IRB) of Kyungpook National University Chilgok Hospital (IRB No. 2023-07-023). This study was conducted in accordance with the Declaration of Helsinki. Written informed consent was obtained from the patients and guardians.

## 3 Results

### 3.1 Baseline characteristics

A total of 146 patients were included in the analysis, with 115 patients in the 2l PEG/Asc group and 31 in the OST group. The median age of patients in PEG/Asc and OST groups was 13.8 (11.5–15.8) and 14.5 (12.9–15.6) years, respectively. The 2l PEG/Asc group included 76 male and 38 female patients, whereas the OST group included 21 male and 10 female patients. Patient weight and body mass index were significantly higher in the OST group than in the 2l PEG/Asc group (Table 1). All patients examined in this analysis were inpatients.

**Table 1:** Baseline characteristics of the patients.

Characteristic	Treatment group		*P*-value
	2l PEG/Asc [*n* = 115]	OST [*n* = 31]	
Age (years)	13.8 [11.5; 15.8]	14.5 [12.9; 15.6]	0.107
Male sex	76 (66.1%)	21 (67.7%)	1.0
Body weight (kg)	46.1 [36.1; 60.4]	53.3 [45.8; 64.4]	0.012
Height (cm)	156.2 ± 15.2	160.9 ± 12.0	0.111
BMI (kg/m^2^)	18.4 [15.9; 21.6]	20.9 [19.1; 23.3]	0.004

BMI, body mass index; PEG/Asc, polyethylene glycol/ascorbate; OST, oral sulfate tablet.

### 3.2 Bowel preparation and procedural outcomes

**Figure 1:**
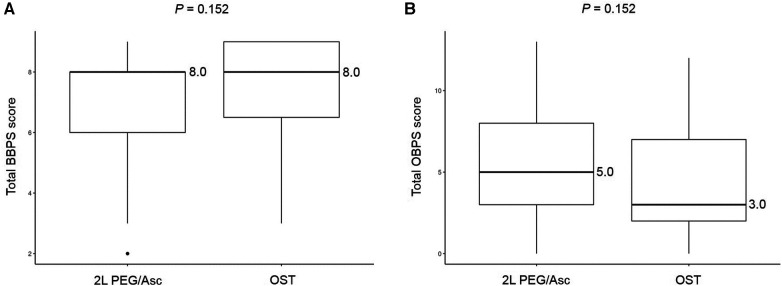
Bowel prepration scales, (**A**) BBPS and (**B**) OBPS, according to the groups. BBPS, Boston bowel preparation scale; OBPS, Ottawa bowel preparation scale; PEG/Asc, polyethylene glycol/ascorbate; OST, oral sulfate tablet.

**Figure 2:**
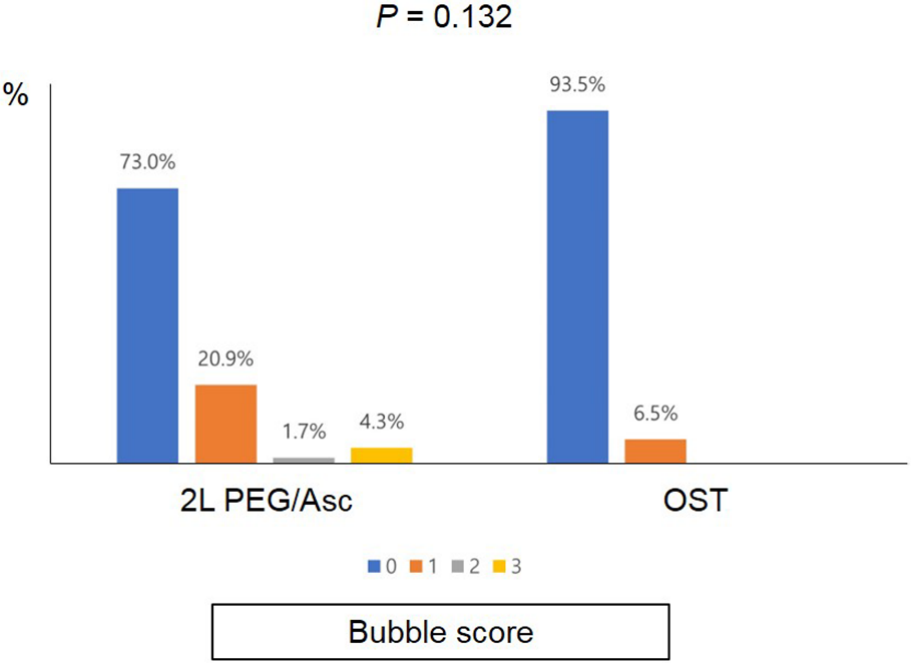
Bubble scores according to the groups. PEG/Asc, polyethylene glycol/ascorbate; OST, oral sulfate tablet.

No significant difference was observed in the total BBPS score [median 8.0 (IQR 6.0–8.0) vs. median 8.0 (IQR 6.5–9.0), *P* = 0.152] and the total OBPS score [median 5.0 (IQR 3.0–8.0) vs. median 3.0 (IQR 2.0–7.0), *P* = 0.152] between the two groups (Figure 1). Based on the BBPS score of ≥6, the rate of adequate bowel preparation was 87.0% (100/115) and 87.1% (27/31) in 2l PEG/Asc and OST groups, respectively. The cecum was reached in 92.2% (106/115) of patients in the 2l PEG/Asc group and 96.8% (30/31) of patients in the OST group. The ratio of patients with a bubble score of 0 was higher in the OST group than in the 2l PEG/Asc group; however, no statistically significant difference was noted between the two groups (73.0% vs. 93.5%, *P* = 0.132) (Figure 2).

### 3.3 Adverse events and safety

**Figure 3:**
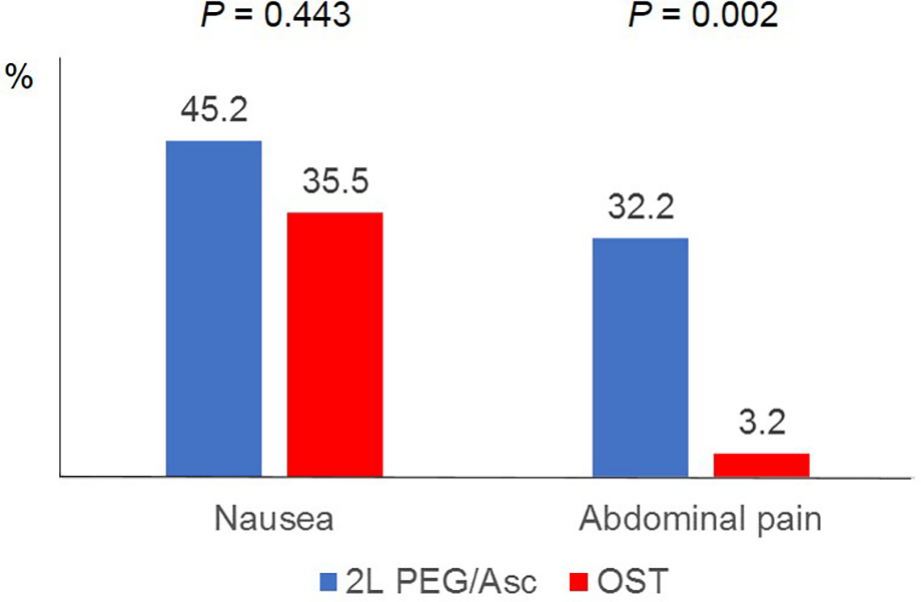
Adverse events according to the groups. PEG/Asc, polyethylene glycol/ascorbate; OST, oral sulfate tablet.

In both groups, the most common adverse event was nausea; however, no statistically significant difference was observed between the two groups (2l PEG/Asc group: 45.2% vs. OST group: 35.5%, *P* = 0.443). Abdominal pain was significantly lower in the OST group (32.2% vs. 3.2%, *P* = 0.002) (Figure 3). In the OST group, only one case complained of abdominal pain. Complaints of vomiting, abdominal distension, dizziness, and thirst were not significantly different between the two groups (Table 2). No significant differences in electrolyte levels and kidney function tests were observed before and after bowel preparation between the two groups, except for chloride levels after bowel preparation. Serum chloride levels were significantly lower in the OTP group compared to the 2l PEG/Asc group [median 105 mEq/L (IQR 104–107) vs. median 107 mEq/L (IQR 105–108), *P* = 0.002] (Table 3).

**Table 2:** Adverse events and tolerability between the 2l PEG/Asc and OST groups.

	Treatment group		*P*-value
	2l PEG/Asc	OST	
Adverse event, *n* [%]			
Nausea	52 (45.2%)	11 (35.5%)	0.443
Vomiting	38 (33.0%)	6 (19.4%)	0.210
Abdominal pain	37 (32.2%)	1 (3.2%)	0.002
Abdominal distension	11 (9.6%)	1 (3.2%)	0.462
Dizziness	6 (5.2%)	1 (3.2%)	1.000
Thirsty	5 (4.3%)	1 (3.2%)	1.000
Tolerability			
Easy-to-drink			<0.001
Very easy	1 (0.9%)	3 (9.7%)	
Easy	13 (11.3%)	15 (48.4%)	
Moderate	26 (22.6%)	6 (19.4%)	
Difficult	47 (40.9%)	6 (19.4%)	
Very difficult	28 (24.3%)	1 (3.2%)	
Taste			0.001
Very good	0 (0%)	0 (0%)	
Good	10 (8.7%)	1 (3.2%)	
Moderate	37 (32.2%)	22 (71.0%)	
Bad	41 (35.7%)	3 (9.7%)	
Very bad	27 (23.5%)	5 (16.1%)	
Overall satisfaction, VAS	4.0 [2.0; 5.0]	7.0 [5.0; 8.0]	<0.001
Willingness to repeat, *n* [%]	39 (33.9%)	26 (83.9%)	<0.001

OST, oral sulfate tablet; PEG/Asc, polyethylene glycol/ascorbate; VAS, visual analog scale.

**Table 3:** Changes in serum electrolyte levels and renal function test results between the 2l PEG/Asc and OST groups.

	Baseline			After preparation	OST	*P*-value
	2l PEG/Asc	OST	*P*-value	2l PEG/Asc		
Serum electrolyte						
Sodium (mEq/L)	137.0 [135.0; 138.0]	138.0 [136.0; 138.0]	0.197	137.9 ± 1.9	138.4 ± 1.6	0.212
Potassium (mEq/L)	4.0 [3.9; 4.3]	4.0 [3.9; 4.2]	0.313	4.1 ± 0.3	4.1 ± 0.4	0.916
Chloride (mEq/L)	106.0 [104.0; 107.0]	105.0 [103.5; 106.0]	0.296	107.0 [105.0; 108.0]	105.0 [104.0; 107.0]	0.002
Magnesium (mg/dl)	2.0 ± 0.2	2.0 ± 0.2	0.080	1.9 ± 0.1	2.0 ± 0.1	0.447
Phosphate (mg/dl)	4.6 ± 0.7	4.6 ± 0.6	0.875	4.7 ± 0.7	4.7 ± 0.9	0.780
Calcium (mg/dl)	9.3 [9.0; 9.7]	9.3 [9.1; 9.6]	0.967	9.4 [ 9.0; 9.7]	9.5 [ 9.3; 9.6]	0.341
Renal function						
Blood urea nitrogen (mg/dl)	10.7 [8.8; 12.8]	12.0 [8.9; 13.6]	0.235	7.7 [6.5; 9.6]	8.9 [7.3; 10.8]	0.067
Creatinine (mg/dl)	0.6 [0.5; 0.7]	0.6 [0.5; 0.7]	0.734	0.6 [0.5; 0.7]	0.6 [0.5; 0.7]	0.398

Data are expressed as means ± standard deviation for continuous variables that showed normal distribution and as median (interquartile range) for continuous variables that did not show normal distribution, unless otherwise indicated.

### 3.4 Tolerability and ease-of-use

**Figure 4:**
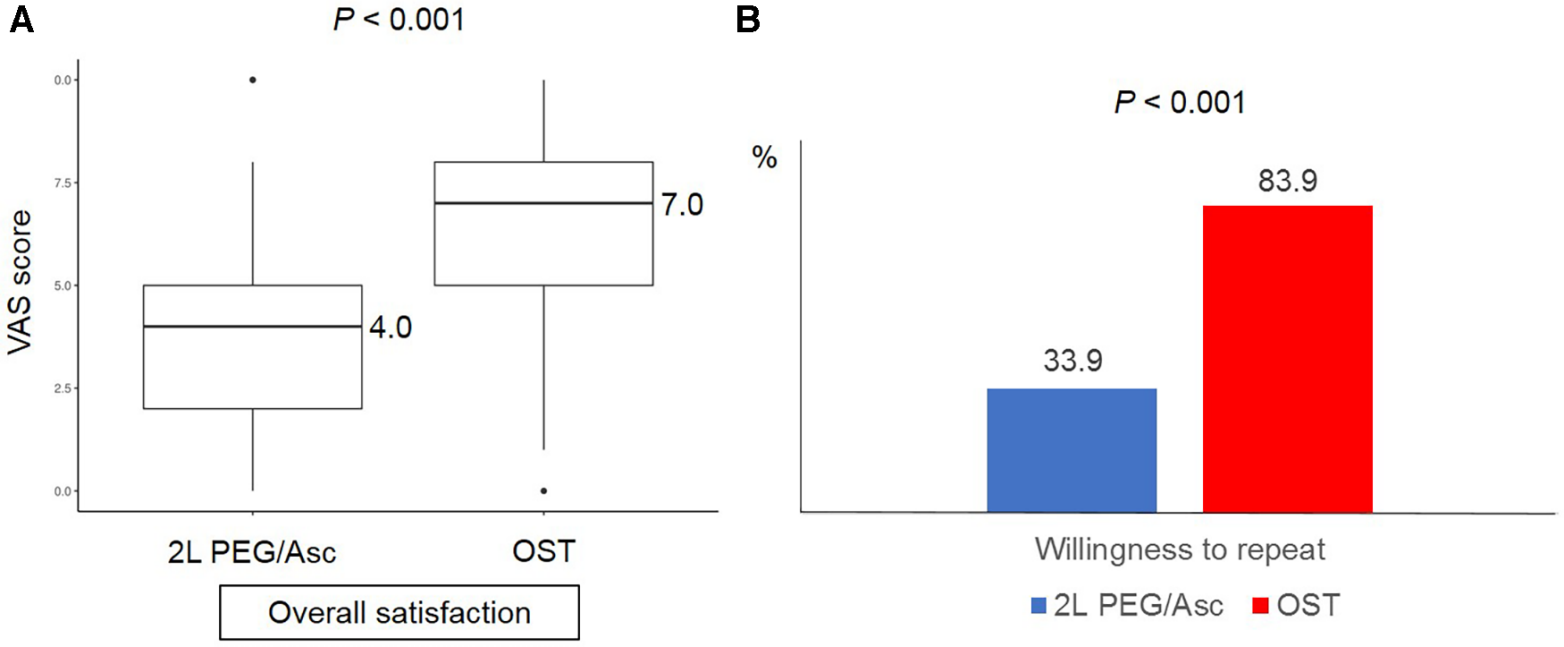
(**A**) Overall satisfaction and (**B**) willingness to repeat according to the groups. VAS, visual analog scale; PEG/Asc, polyethylene glycol/ascorbate; OST, oral sulfate tablet.

Complete ingestion of bowel preparation regimens was achieved in 71.3% (82/115) and 87.1% (27/31) of patients in 2l PEG/Asc and OST groups, respectively. Three patients in the 2l PEG/Asc group required NG tube placement for the completion of bowel preparation. No patient in the OST group required NG tube insertion. The ease-to-drink score, which was answered as easy or very easy, was significantly higher in the OST group (12.2% vs. 58.1%, *P* < 0.001). The taste score (answered as bad or very bad) was significantly lower in the OST group than in the 2l PEG/Asc group (59.1% vs. 25.8%, *P* = 0.001). The VAS score for overall satisfaction was significantly higher in the OST group than in the 2l PEG/Asc group (4.0 vs. 7.0, *P* < 0.001) (Figure 4). For the next colonoscopy bowel preparation, a higher proportion of patients in the OST group showed a willingness to use the same preparation regimen (33.9% vs. 83.9%, *P* < 0.001) (Table 2).

## 4 Discussion

Compared with adults, children have limited experience with ingestion of OST for bowel preparation in colonoscopy. To the best of our knowledge, this is the first study to analyze the efficacy and safety of OST for bowel preparation in children.

To date, OST has not yet been universally approved for use in children. When the administration of a previously approved bowel preparation regimen is challenging, OST is considered a secondary option based on the voluntary intention of patients and guardians. In studies involving adults, the use of OST for bowel preparation was not inferior in efficacy and was safe and well tolerated (15–17).

Suboptimal bowel preparation is common in children. Previous studies have shown that bowel preparation is inadequate in up to 30% of children (7–10). Recently, the American Society for Gastrointestinal Endoscopy suggested that the minimum target for the key quality indicator “rate of adequate bowel preparation” should be ≥80% for pediatric colonoscopy (18, 19). In the present study, 87.0% of patients in the 2l PEG/Asc group and 87.1% of them in the OST group demonstrated adequate bowel preparation based on the BBPS score.

Many bowel preparation scales have been developed and analyzed in adult colonoscopy, including BBPS and OBPS (20, 21). This study showed that the results of the bowel preparation scales were not significantly different between the two groups. Although these scoring systems are usually employed in pediatrics, they have not yet been systematically validated in children. Thus, it is necessary to develop a bowel preparation scale specialized for children.

The recent European Society of Gastrointestinal Endoscopy (ESGE) guideline suggested the addition of oral simethicone, an antifoaming agent, to the bowel preparation (22). Minimizing foaming is important for high-quality colonoscopy. In our OST group, the proportion of patients who achieved a bubble score of 0 was as high as 93%. Because OST contains simethicone, patients who administer OST can demonstrate excellent bubble elimination without additional administration of simethicone.

Patient acceptance is of utmost importance in pediatric bowel preparation. According to ESGE/European Society for Pediatric Gastroenterology Hepatology and Nutrition (ESGE/ESPGHAN) guideline, low-volume preparation using PEG along with Asc or picosulfate magnesium citrate is recommended for bowel preparation in children (6). Pediatric patients who strongly refuse to administer a bowel preparation solution will inevitably require NG tube insertion. In the OST group, NG tube insertion was not needed, and the scores of ease-of-use, taste, and intention to reuse at the next colonoscopy were significantly better than those in the 2l PEG/Asc group. Although patients in both groups ingested the same amount of liquid, those in the OST group only needed to drink plain water. Currently, the Food and Drug Administration has prohibited the use of NaP in children (7); however, previous studies in adults have shown that NaP tablets were better tolerated than solution-based NaP (12–14).

Although tablets are relatively easy to administer, children often complain of difficulty taking pills. In this study, the complete ingestion rate of the OST group was 87.1%, whereas that of adults in the OST group was 100% in a previous study (16). In addition, all patients in our study underwent bowel preparation and colonoscopy during hospitalization, which leads to problems such as school absences in school-age patients. Therefore, a bowel preparation method that promotes compliance in children and has minimal effect on daily life is desirable.

In our study, OST was safe for use in bowel preparation in children, and no serious adverse events were reported. Abdominal pain was reported in 3.2% of patients in the OST group, which was significantly lower than that in the 2l PEG/Asc group (32.2%). Other clinical symptoms related to adverse events were not significantly different between the two groups. In both groups, laboratory values including serum electrolyte levels and renal function test results revealed no significant changes after bowel preparation and were within normal ranges.

This study has several limitations. First, this was a retrospective study with a small sample size and was conducted at a single children's hospital. Multicenter prospective studies on pediatric bowel preparation are warranted. Second, selection bias may have occurred; only 31 patients used OST for bowel preparation. OST has not yet been universally approved in children. Therefore, when selecting regimens, both the preference of the bowel preparation agent as well as issue of licensing must be considered.

Despite these limitations, this is the first study to evaluate the efficacy and safety of OST for pediatric bowel preparation. Although there is an increasing need for pediatric colonoscopy, no protocol for pediatric bowel preparation has been universally established. Bowel preparation in children presents many challenges. To improve intake compliance, tableted purgatives can be considered a good alternative in children. This study showed that OST was not inferior to 2 L of PEG/Asc in terms of bowel cleansing efficacy and safety but was associated with superior patient compliance.

In conclusion, OST was as efficacious and safe as 2 L of PEG/Asc for bowel preparation in children. It is important to improve patient acceptance in children. Regarding tolerability, OST was more satisfactory than 2 L of PEG/Asc. OST can be considered a good alternative for children who have poor compliance during bowel cleansing.

## Data Availability

The original contributions presented in the study are included in the article/Supplementary Material, further inquiries can be directed to the corresponding author.

## References

[1] Park JH. Pediatric colonoscopy: the changing patterns and single institutional experience over a decade. Clin Endosc. (2018) 51:137–41. 10.5946/ce.2018.051 29618177 PMC5903084

[2] Hong SJ Cho SM Choe BH Jang HJ Choi KH Kang B Characteristics and incidence trends for pediatric inflammatory bowel disease in Daegu-Kyungpook province in Korea: a multi-center study. J Korean Med Sci. (2018) 33:e132. 10.3346/jkms.2018.33.e132 29713253 PMC5920122

[3] Lee SW Kang B Choi S Choe BH Kim YB Lee KJ The changes in trends of lower gastrointestinal endoscopy conducted in children and adolescents after the COVID-19 outbreak in Korea. Medicina (Kaunas). (2022) 58:1378. 36295539 10.3390/medicina58101378 PMC9608561

[4] Choe JY Choi S Song KH Jang HJ Choi KH Yi DY Incidence and prevalence trends of pediatric inflammatory bowel disease in the Daegu-Kyungpook province from 2017 to 2020. Front Pediatr. (2022) 9:810173. 10.3389/fped.2021.810173 35059365 PMC8764442

[5] Lee YM Lee Y Choi SY Kim HJ Hong SJ Kang Y A nationwide survey on gastrointestinal endoscopy practice patterns among pediatric endoscopists in South Korea. Pediatr Gastroenterol Hepatol Nutr. (2023) 26:79–87. 10.5223/pghn.2023.26.2.79 36950059 PMC10025574

[6] Thomson M Tringali A Dumonceau JM Tavares M Tabbers MM Furlano R Paediatric gastrointestinal endoscopy: European society for paediatric gastroenterology hepatology and nutrition and European society of gastrointestinal endoscopy guidelines. J Pediatr Gastroenterol Nutr. (2017) 64:133–53. 10.1097/MPG.0000000000001408 27622898

[7] Pall H Zacur GM Kramer RE Lirio RA Manfredi M Shah M Bowel preparation for pediatric colonoscopy: report of the NASPGHAN endoscopy and procedures committee. J Pediatr Gastroenterol Nutr. (2014) 59:409–16. 10.1097/MPG.0000000000000447 24897169

[8] Dahshan A Lin CH Peters J Thomas R Tolia V. A randomized, prospective study to evaluate the efficacy and acceptance of three bowel preparations for colonoscopy in children. Am J Gastroenterol. (1999) 94:3497–501. 10.1111/j.1572-0241.1999.01613.x 10606310

[9] Kumar S Bennett WE Bozic MA Croffie JM Ferrell E Hon EC Inadequate bowel preparation in pediatric colonoscopy-prospective study of potential causes. J Pediatr Gastroenterol Nutr. (2021) 73:325–8. 10.1097/MPG.0000000000003178 34415261

[10] Reddy P Mencin A Lebwohl B. Risk factors for suboptimal bowel preparation for colonoscopy in pediatric patients. J Pediatr Gastroenterol Nutr. (2021) 73:e1–6. 10.1097/MPG.0000000000003114 33661246

[11] Gordon M Karlsen F Isaji S Teck GO. Bowel preparation for elective procedures in children: a systematic review and meta-analysis. BMJ Paediatr Open. (2017) 1:e000118. 10.1136/bmjpo-2017-000118 29637141 PMC5862165

[12] Aronchick CA Lipshutz WH Wright SH Dufrayne F Bergman G. A novel tableted purgative for colonoscopic preparation: efficacy and safety comparisons with colyte and fleet phospho-soda. Gastrointest Endosc. (2000) 52:346–52. 10.1067/mge.2000.108480 10968848

[13] Johanson JF Popp JW Jr Cohen LB Lottes SR Forbes WP Walker K A randomized, multicenter study comparing the safety and efficacy of sodium phosphate tablets with 2l polyethylene glycol solution plus bisacodyl tablets for colon cleansing. Am J Gastroenterol. (2007) 102:2238–46. 10.1111/j.1572-0241.2007.01363.x 17573796

[14] Kastenberg D Chasen R Choudhary C Riff D Steinberg S Weiss E Efficacy and safety of sodium phosphate tablets compared with PEG solution in colon cleansing: two identically designed, randomized, controlled, parallel group, multicenter phase III trials. Gastrointest Endosc. (2001) 54:705–13. 10.1067/mge.2001.119733 11726845

[15] Yang HJ Park DI Park SK Lee CK Kim HJ Oh SJ Novel sulfate tablet PBK-1701TC versus oral sulfate solution for colon cleansing: a randomized phase 3 trial. J Gastroenterol Hepatol. (2020) 35:29–36. 10.1111/jgh.14826 31396995

[16] Kim KO Kim EY Lee YJ Lee HS Kim ES Chung YJ Efficacy, safety and tolerability of oral sulphate tablet for bowel preparation in patients with inflammatory bowel disease: a multicentre randomized controlled study. J Crohns Colitis. (2022) 16:1706–13. 10.1093/ecco-jcc/jjac080 35689818

[17] Di Palma JA Bhandari R Cleveland MV Mishkin DS Tesoriero J Hall S A safety and efficacy comparison of a new sulfate-based tablet bowel preparation versus a PEG and ascorbate comparator in adult subjects undergoing colonoscopy. Am J Gastroenterol. (2021) 116:319–28. 10.14309/ajg.0000000000001020 33165006 PMC7864663

[18] Walsh CM Lightdale JR. Pediatric endoscopy quality improvement network (PEnQuIN) quality standards and indicators for pediatric endoscopy: an ASGE-endorsed guideline. Gastrointest Endosc. (2022) 96:593–602. 10.1016/j.gie.2022.06.016 36028336

[19] Lightdale JR Walsh CM Oliva S Jacobson K Huynh HQ Homan M Pediatric endoscopy quality improvement network quality standards and indicators for pediatric endoscopic procedures: a joint NASPGHAN/ESPGHAN guideline. J Pediatr Gastroenterol Nutr. (2022) 74:S30–43. 10.1097/MPG.0000000000003264 34402486

[20] Kastenberg D Bertiger G Brogadir S. Bowel preparation quality scales for colonoscopy. World J Gastroenterol. (2018) 24:2833–43. 10.3748/wjg.v24.i26.2833 30018478 PMC6048432

[21] Parmar R Martel M Rostom A Barkun AN. Validated scales for colon cleansing: a systematic review. Am J Gastroenterol. (2016) 111:197–204; quiz 205. 10.1038/ajg.2015.417 26782820

[22] Hassan C East J Radaelli F Spada C Benamouzig R Bisschops R Bowel preparation for colonoscopy: European society of gastrointestinal endoscopy (ESGE) guideline—update 2019. Endoscopy. (2019) 51:775–94. 10.1055/a-0959-0505 31295746

